# A Mobile App for Wound and Symptom Surveillance After Colorectal Surgery: Protocol for a Feasibility Randomized Controlled Trial

**DOI:** 10.2196/26717

**Published:** 2022-01-14

**Authors:** Heather Anne Valk, Carlos Garcia-Ochoa, Jessica Fontaine Calder, Toba Miller, Babak Rashidi, Corrine McIsaac, Reilly Musselman

**Affiliations:** 1 Division of General Surgery Faculty of Medicine University of Ottawa Ottawa, ON Canada; 2 ARC Innovation Center Ottawa Hospital Research Institute Ottawa, ON Canada; 3 Wound, Ostomy, and Rehabilitation The Ottawa Hospital Ottawa, ON Canada; 4 Department of Internal Medicine Faculty of Medicine University of Ottawa Ottawa, ON Canada; 5 Health Outcomes Worldwide Toronto, ON Canada

**Keywords:** eHealth, mobile app, surgical site infection, colorectal surgery, app, surgery, infection, wound, surveillance, feasibility, randomized controlled trial, tracking, patient experience, COVID-19, transmission

## Abstract

**Background:**

Surgical site infections (SSIs) are the most common nosocomial infection and occur in 16.3% of patients undergoing colorectal surgery at our institution (The Ottawa Hospital), the majority of which are identified after discharge from hospital. Patients who suspect having an SSI generally present to the emergency department or surgery clinic. Both options for in-person interaction are costly to the health care system and patients. A mobile app, how2trak, has proven to be beneficial for patients with complex wounds at our institution by facilitating at-home monitoring and virtual consultations.

**Objective:**

This study aims to assess the feasibility of a randomized controlled trial to assess if how2trak can improve patients’ experience and increase detection of SSIs after colorectal surgery while reducing patients’ risk of COVID-19 exposure.

**Methods:**

In this single-center prospective feasibility trial, eligible patients undergoing colorectal surgery will be randomized to either standard care or how2trak postoperative monitoring of their incision, symptoms, and ostomy function. Patient self-assessments will be monitored by a nurse specialized in wound and ostomy care who will follow-up with patients with a suspected SSI. The primary outcome is feasibility as measured by enrollment, randomization, app usability, data extraction, and resource capacity.

**Results:**

This study was approved by our institution’s ethics board on February 26, 2021, and received support from The Ottawa Hospital Innovation and Care Funding on November 12, 2021. Recruitment started June 3, 2021, and 29 were patients enrolled as of September 2021. We expect to publish results in spring 2022.

**Conclusions:**

This study will determine the feasibility of using a mobile app to monitor patients’ wounds and detect SSIs after colorectal surgery. If feasible, we plan to assess if this mobile app facilitates SSI detection, enhances patient experience, and optimizes their care.

**Trial Registration:**

ClinicalTrials.gov NCT04869774; https://clinicaltrials.gov/ct2/show/NCT04869774

**International Registered Report Identifier (IRRID):**

DERR1-10.2196/26717

## Introduction

### Background and Rationale

Surgical site infections (SSIs) are the most common nosocomial infection among surgical patients, occurring in 5.5% of surgical cases at our institution, The Ottawa Hospital [1]. Similar rates are seen at other centers across North America and Europe, with SSIs impacting 3%-5% of inpatient surgical cases and accounting for 20%-38% of nosocomial infections among surgical patients [2-5]. Patients undergoing colorectal surgery are disproportionately affected. A recent study at our institution found that 24% of patients undergoing colorectal surgery developed an SSI [6]. SSIs are associated with significant morbidity and mortality both in the short and long term. In the initial postoperative period, SSIs are associated with increased postoperative mortality, length of stay, and intensive care unit admission. In the long term, SSIs are independently associated with higher readmission and mortality rates compared to those without an SSI [1].

### SSIs and a Need for Postoperative Surveillance

Approximately two-thirds of SSIs are defined as superficial, meaning they involve only the skin and subcutaneous tissues [5]. These SSIs are detectable by clinical exam; when identified early, they can be managed with wound management alone. However, approximately 57% of SSIs are diagnosed after discharge from hospital, when patients are no longer regularly examined by a clinician [7]. When comparing patients with SSIs detected after discharge from hospital to those with SSIs detected in hospital, the former are more likely to have an advanced infection at time of detection and experience poorer quality of life, greater physical limitations, and lower mental health component scores, as well as have increased outpatient and emergency department (ED) visits, hospital readmissions, and higher demand for diagnostic imaging [8,9]. The incidence and morbidity of an SSI can be reduced by regular clinical surveillance of the incision [10].

### Mobile App Technology

Mobile apps are an effective and desirable method for assisting patients with self-monitoring for SSIs and other complications after surgery [11]**.** Photo-based mobile apps have been shown in multiple studies to be effective at detecting superficial SSIs [12-14]. This can also reduce a patient’s need for in-person visits by facilitating virtual monitoring and virtual visits with a clinician. This is critical in the context of the COVID-19 pandemic, and could reduce ED visits and readmission in the postoperative period [15]. Finally, the postoperative period can be stressful and isolating for patients. Patients using mobile apps for surveillance after surgery have reported a greater sense of empowerment, as well as more engagement and satisfaction [16].

The Health Outcomes Worldwide how2trak (H2T) app is an example of a mobile point-of-care technology for wound surveillance. H2T has been shown to be an effective tool for postoperative monitoring after discharge from hospital following a cesarean delivery. However, this app has not been assessed among colorectal surgery patients, who represent a more diverse, comorbid, and older population than young females undergoing cesarean delivery [17]. H2T has been adopted at The Ottawa Hospital for wound monitoring in the vascular surgery patient population, and plans are underway to integrate it with the hospital electronic medical record system. H2T could potentially enhance postoperative monitoring and detection of superficial SSI but would require a significant change in workflow to the multidisciplinary care of wound complications. Therefore, its feasibility must be assessed prior to conducting a trial to assess its effectiveness in this population.

### Objectives

This study aims to assess the feasibility of a randomized controlled trial where patients discharged from hospital after colorectal surgery are randomized to have virtual monitoring of their surgical incision(s), symptoms, and ostomy using the H2T app or to standard of care. The primary outcome is feasibility as measured by enrollment, randomization, H2T usability, data extraction, and resource capacity.

This is intended to inform the development of a definitive trial to establish if the H2T app enhances SSI detection, improves patients’ experience, and reduces the risk of COVID-19 transmission compared to standard care.

## Methods

### Overview

This protocol was developed in accordance with the SPIRIT (Standardized Protocol Items: Recommendations for Interventional Trials) statement [18] and CONSORT-EHEALTH (Consolidated Standards of Reporting Trials of Electronic and Mobile Health Applications and Onine Telehealth) reporting recommendations [19]. This study has been approved by the Research Ethics Board at the Ottawa Hospital Research Institute (20200596-01H).

### Study Setting

This study will be conducted with the Ottawa Colorectal Group within the Division of General Surgery at The Ottawa Hospital, a multisite academic hospital in Ottawa, Ontario, Canada.

### Trial Design

This is an unblinded feasibility randomized controlled trial. Eligible patients will be randomized 1:1 to receive virtual monitoring of their surgical incision, symptoms, and ostomy using H2T or to standard of care.

### Eligibility Criteria

All patients being discharged from hospital after undergoing urgent, semiurgent, or elective abdominal surgery by a colorectal surgeon at The Ottawa Hospital will be considered for participation in the study.

To be included in the study, participants must be ≥16 years of age, patients who are being discharged from hospital after undergoing semiurgent, urgent, or elective abdominal surgery by a colorectal surgeon at The Ottawa Hospital, and have provided informed consent to participate. Patients enrolled in other clinical trials will still be candidates for this feasibility trial.

Patients will be excluded if they are <16 years of age, have no access to or ability to use a mobile device, have no cellular data/Wi-Fi access, and/or cannot read and write in English.

Clinicians using the H2T app in this study will be considered study participants as well. They will be asked to complete the Modified Post-Study System Usability Questionnaire (PSSUQ) for Patients and Clinicians, a survey derived from the PSSUQ by Lewis and colleagues [20]. This survey addresses the experience of using the H2T app; their feedback regarding usability is fundamental for future improvement.

### Informed Consent

The colorectal surgery team will notify the research assistant of all patients undergoing urgent, semiurgent, or elective abdominal surgery by a colorectal surgeon at The Ottawa Hospital who are potentially eligible for participation within 72 hours following the patient’s surgery. The research assistant will then meet with patients by phone or in person to screen for eligibility, and proceed with the consent discussion, which will include the following: the nature of the trial, eligibility criteria, and the risks and benefits of participating. The research assistant will also assess their willingness to participate, and explain that their data may be included in a full-scale randomized controlled trial if completed as a vanguard study.

Clinicians who have used H2T during the study period will be contacted by email by the research assistant and will be invited to complete the Patient and Clinician Survey of Application survey. The completion of the survey will indicate their acceptance to participate in the study.

### Interventions

**Figure 1:**
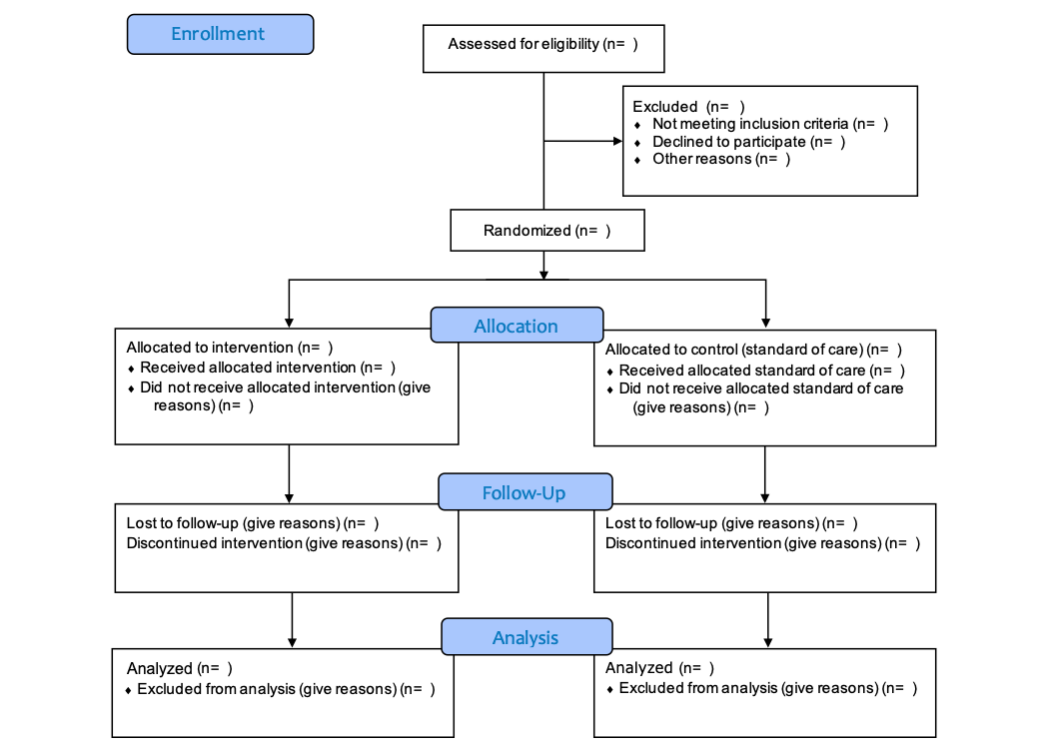
CONSORT flow diagram.

Immediately after consent is obtained, eligible patients will be randomized 1:1 to two arms (Figure 1).

**Figure 2:**
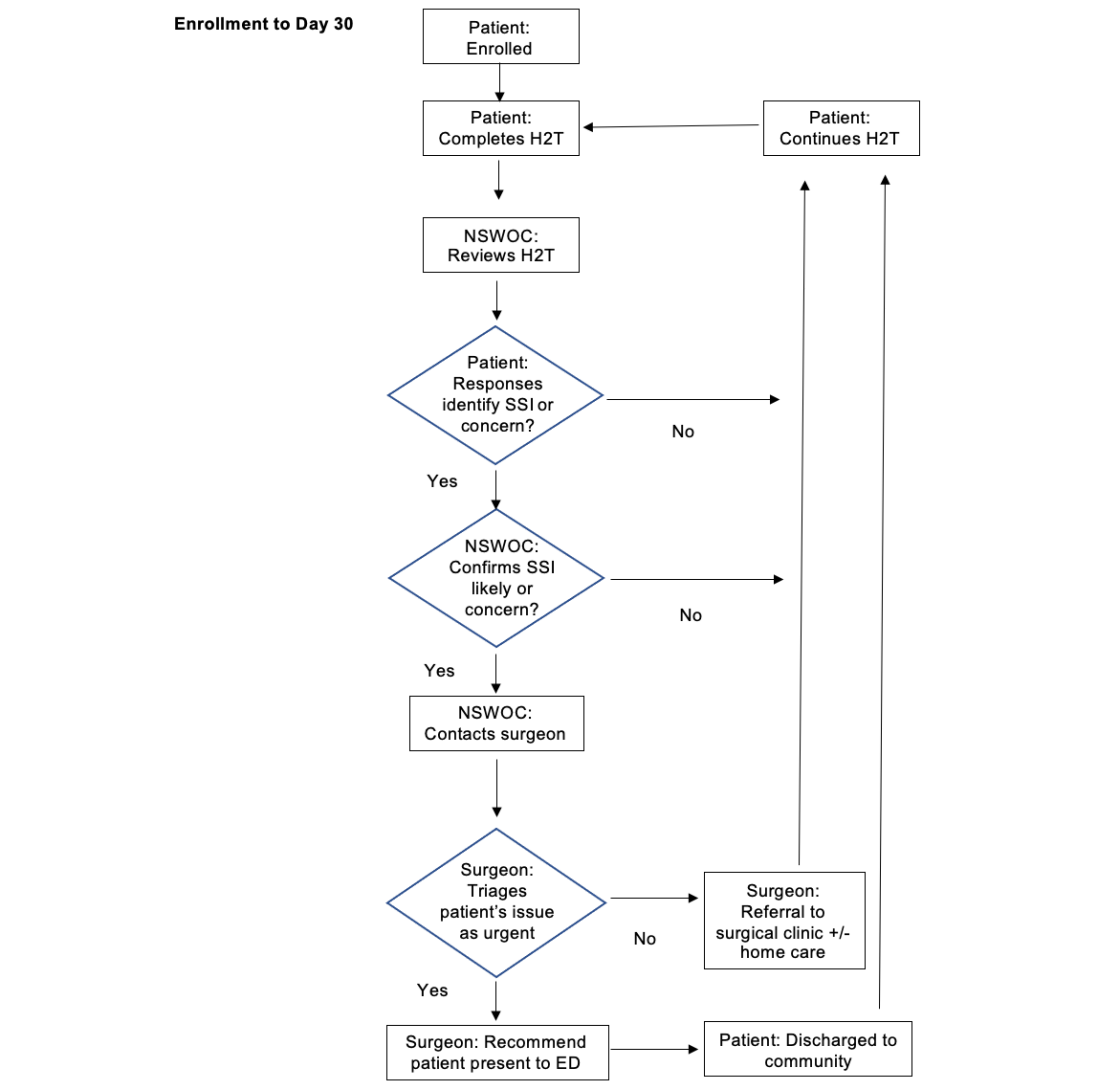
Nurse Specialized in Wound, Ostomy, and Continence (NSWOC) triage. Wound And Symptom Tracking After Colorectal Surgery Using How2trak (WATCH) study NSWOC workflow process. Urgent refers to a concern that requires immediate medical attention. ED: emergency department; H2T: how2trak; SSI: surgical site infection (based on the Centers for Disease Control and Prevention definition, see Patient Application Response suggestive of surgical site infection).

Intervention group patients will undergo virtual monitoring of their incision and symptoms using H2T. Early in their postoperative course, patients will be guided through developing a login and instructed on how to download and use H2T by the research assistant. In developing their login, patients will enter their full name and partial date of birth (MM-YYYY), which will be used as identifiers to link to The Ottawa Hospital data. Using the H2T app, patients will be asked to answer a series of questions (see Patient Application Questionnaire in Multimedia Appendix 1) and photograph their surgical incision on postoperative day 3, 5, 7, 10, 20, and 30 through the app (see timeline in Table 1). Patient responses and photographs entered into the H2T app on the patient’s mobile device will be reviewed within 72 hours by a trained Nurse Specialized in Wound, Ostomy, and Continence (NSWOC) [21] who will be trained to triage patients using the algorithm in Figure 2. If a concern is identified, the NSWOC will contact the patient to arrange a virtual visit using the H2T app or notify the surgery team (including colorectal surgeons and physician residents) in accordance with the algorithm, clinical discretion, and scope of practice. Patients will be reminded that it may take up to 72 hours to receive a response from the NSWOC; accordingly, if they have urgent concerns or emergency, they should present to the ED or call 911.

Control group patients will receive no virtual monitoring. No virtual monitoring is currently in place as part of standard of care after colorectal surgery at The Ottawa Hospital. Therefore, standard of care without any virtual monitoring was selected as the comparator.

Both groups will receive routine standard of care. Standard of care will involve a routine postoperative follow-up with the surgical team 4-6 weeks after discharge from hospital.

Patients and clinicians will complete the PSSUQ, a validated 16-item survey to assess the perceived learnability, efficiency, and errors of H2T, as well as user satisfaction with the app [20,22] (see Multimedia Appendix 1). Patients will also submit a survey to assess their satisfaction with their postoperative care at postoperative day 30 to assess their perceived experience of their postdischarge care using a modified version of PATSAT32, a validated questionnaire for patients undergoing gastrointestinal surgery [23,24] (see Multimedia Appendix 1). The intervention will be reviewed every two months by the investigator team and General Surgery Comprehensive Unit-based Safety Program team. H2T functionality will remain consistent throughout the study, with revisions made only in response to unexpected events.

**Table 1:** Participant timeline.

Timepoint		Study period									
		Visit with research assistant	Visit with research assistant	Intervention period (postoperative day 3-30)							Close-out
		–*t*_1_	0-3	3	5	7	10	20	30	*t*_x_	
**Enrolment**											
	Eligibility screen	✓	✓								
	Informed consent	✓	✓								
	Allocation		✓								
**Interventions**											
	How2Trak virtual monitoring			✓	✓	✓	✓	✓	✓	✓	
	Control								✓	✓	
**Assessments**											
	Baseline comorbidities	✓	✓								
	Health system interaction			✓	✓	✓	✓	✓	✓	✓	
	Postoperative outcomes			✓	✓	✓	✓	✓	✓	✓	
	Patient experience								✓	✓	
	App usability									✓	

### Strategies to Improve Adherence to the Intervention

Patients will receive notifications through the app to serve as a reminder each day that a photo and questionnaire are required. If no response is received, the research assistant will follow up with the patient by email and/or by phone. In keeping with the pragmatic nature of this trial, patients may request to withdraw from the intervention group at any time.

### Outcomes

#### Primary Outcome: Feasibility

This study will assess the feasibility of a definitive trial by assessing specific outcome measures that will be determined to be feasible if all endpoints are met:

1. Capability for enrollment will be assessed to determine recruitment potential and an optimal sample size estimation for a full-scale randomized controlled trial, by measuring the following outcomes: proportion of patients screened, proportion of eligible patients consenting to involvement in this study, and proportion of recruited patients who are enrolled in the study.
  - Endpoint: 4 patients per month on average are enrolled in the study.
2. Feasibility of the randomization processes will be evaluated, including the proportion of patients randomized who receive the intervention.
  - Endpoint: 90% of patients enrolled are randomized to the intervention or control group.
3. How2trak usability, delivery, and compliance will be evaluated by assessing patients’ and health care providers’ perceived usability of the technology, as well as the number of questionnaires and photos completed by each patient during the postoperative period.
  - Endpoints: Patients completed, on average, 60% of self-assessments. The mean score of the H2T app in patient and clinician surveys is >2.
4. Feasibility of data extraction, measured by the proportion of participants for which all relevant outcomes of a full-scale randomized controlled trial were documented.
  - Endpoint: All primary outcomes of the definitive trial (see “Definitive Trial Outcomes” section below) are recorded for 80% of patients.
5. Resources and time required to conduct the feasibility trial will be assessed. In particular, the administrative capacity, expertise, skills, space, and time of the research team, the feasibility of the designated budget, and the compliance of interdisciplinary staff with the study protocol will be assessed.
  - Endpoint: The feasibility study is completed with the allocated interdisciplinary staff.

#### Definitive Trial Outcomes

The primary outcomes for a definitive trial collected in this study will compare the SSI incidence and severity of patients in the two study arms, as well as their experiences. An SSI is an infection that occurs after surgery in the part of the body where the surgery was performed. A superficial incisional SSI is an infection that involves only skin or subcutaneous tissue of the surgical incision and will be defined using the Centers for Disease Control and Prevention criteria [5].

Secondary outcomes for a definitive trial include hours of in-person interactions with the health care system (including readmissions to hospital, ED visits, and clinic visits), confirmed COVID-19 infection within 30 days after surgery, incidence of postoperative adverse events, and incidence and characterization of ostomy complications.

### Statistical Analysis

Descriptive statistical analysis will be conducted using Microsoft Excel (version 16.16.19; Microsoft Corp) and RStudio (version 1.1463).

For the definitive trial, the following tests will be used to discern relationships between variables: two-sample Welch *t* test for normally distributed continuous variables, Kruskal-Wallis rank-sum test for data without normal distribution, and chi-square goodness of fit test for categorical variables. A *P* value of <.05 will be considered statistically significant.

### Sample Size

There is no sample size for this feasibility study. It will be conducted over 6 months and we anticipate having 80 eligible patients over this time period.

### Assignment of Interventions

After written informed consent is obtained, eligible patients will be randomly assigned to either the intervention or control group using a secure web-based randomization system at the Methods Centre of The Ottawa Hospital Research Institute. This study will be unblinded so no attempts at concealment of patients’ allocation will be made.

### Data Collection and Management

Data will be captured through the assessments and/or photographs in H2T and the hospital’s electronic health information system (Epic). Data sent from H2T to The Ottawa Hospital—including patient identifiers to allow for merging with Epic data—will be transferred through a secure, access-controlled folder within Office 365. Once the data files are merged, they will be deidentified. Only research personnel will have access to the study data. A data monitoring committee will not be developed given the minimal risk and small sample size of this feasibility trial.

If a definitive trial is completed, we plan to incorporate the results of this study into the data collection of the trial.

### Confidentiality

Data will be originally stored locally on patients’ devices, encrypted, in a secure app sandbox of the H2T app. The H2T app will attempt to synchronize its internal database with the server's database whenever there is a need (ie, new information is created). Once the data are on the server, they will be retained with patient identifying information intact for a length of time (such as 6 or 10 years), as determined by the requirements of the jurisdiction in which the data is being collected.

The Ottawa Hospital data will be collected primarily from Epic using Workbench reports. Data from Health Outcomes Worldwide’s encrypted servers will be securely transferred to the research team, who will merge it with data collected primarily from Epic using patients’ names and partial dates of birth as identifiers (entered by patients when developing their login). A master list (containing the study ID and patient identifiers) will be accessible only to the researchers responsible for the analysis and will be kept separate from the main study data set in a secure, access-controlled folder on The Ottawa Hospital’s Office 365 cloud. The Ottawa Health Science Network Research Ethics Board (OHSN-REB) and Ottawa Hospital Research Institute may review relevant study records (under the supervision of the investigator) for audit purposes.

### Oversight and Monitoring: Adverse Event Reporting and Harms

Clinicians involved in the trial (surgical team and virtual care NSWOC) will monitor for adverse events and report them immediately to the Principal Investigator. The investigator team will report to the Research Ethics Board any adverse event that is deemed to be unexpected, related or possibly related to the investigational product or other study intervention/treatment/procedure or to participation in the research (ie, definitely, probably, possibly, or unlikely related) and that involves greater risk.

If the adverse event meets reporting criteria, the event will be submitted within seven calendar days of the team becoming aware of the incident using the OHSN-REB Reportable Events form. There are no plans for audits of the trial conduct.

### Management Team

Principal investigators are RM (colorectal surgeon and quality improvement lead at The Ottawa Hospital) and HAS (general surgery resident, clinical investigator program graduate, and resident quality improvement lead). Coinvestigators include CHO (general surgery resident), JFC (Project Manager, ARC Innovation Center, Ottawa Hospital Research Institute), TM (NSWOC and advanced practice nurse at The Ottawa Hospital), and BR (internal medicine physician at The Ottawa Hospital), as well as two patient partners recruited to assist in trial design and analysis of results.

## Results

This study was approved by the OHSN-REB on February 26, 2021, and accepted for The Ottawa Hospital Innovation and Care Funding on November 12, 2021. Recruitment started June 3, 2021, and as of September 2021, we had 29 patients enrolled. We expect to publish results in spring 2022.

## Discussion

We anticipate this work will help us to better understand the feasibility of using mobile technology to optimize patients’ care after discharge from hospital after colorectal surgery. Virtual postsurgery wound and symptom monitoring could enhance the patient experience, SSI detection, and reduce the risk of COVID-19 transmission. If this technology is feasible for our patient population and workflow, we plan to formally assess its effectiveness with a definitive trial. The H2T app has multiple functionalities, and this study could establish a framework for assessing its use in other domains including ostomy monitoring and patient education, as well as for patients undergoing other surgical procedures.

## Appendix

Multimedia Appendix 1 Supplemental materials.

## Abbreviations

CONSORT-EHEALTH: Consolidated Standards of Reporting Trials of Electronic and Mobile Health Applications and Online Telehealth
ED: emergency department
H2T: how2trak
NSWOC: Nurse Specialized in Wound, Ostomy, and Continence
OHSN-REB: Ottawa Health Science Network Research Ethics Board
PSSUQ: Post-Study System Usability Questionnaire
SPIRIT: Standardized Protocol Items: Recommendations for Interventional Trials
