# Supplementary material for: A Mobile App for Wound and Symptom Surveillance After Colorectal Surgery: Protocol for a Feasibility Randomized Controlled Trial
Source: JMIR Res Protoc. 2022 Jan 14;11(1):e26717. doi: 10.2196/26717 (PMC8763310; doi:10.2196/26717)
Supplement: Multimedia Appendix 1 [file resprot_v11i1e26717_app1.docx]

## Supplementary file 1: Patient Application (app) Questionnaire

**INCISION**

**Since the last App assessment**

1. Have you contacted or seen a health care provider because worried about your surgery incision?

YES

Did they change any of your incision treatment (i.e. new bandage, medication)?

Yes

Specify __________

No

NO

1. Is there more redness around your surgery incision?

YES

NO

1. Do you have worse pain around your surgery incision?

YES

Select 0-10 on pain scale (0 – None, 10 – Excruciating): 0 1 2 3 4 5 6 7 8 9 10

NO

1. Is there drainage coming from your surgery incision?

YES

Compared to before, the drainage is:

LESS

MORE

SAME

NONE

1. The smell from your surgery incision is:

NONE

LESS

SAME

STRONGER

**PAIN**

**Since the last App assessment**

1. Are you taking any pain medication for discomfort because of your surgery?

YES

The medication you are taking includes (check all that apply):

Tylenol

Advil

Naproxen

Celebrex

Tramadol

Dilaudid

Morphine

Other (specifiy): ___________

NO

**OSTOMY**

**Since the last App assessment**

1. After leaving the hospital, have you received a home care visit for ostomy care?

YES

NO

1. Have you contacted or seen a healthcare provider because you were worried about your ostomy?

YES

Did they change any of your treatment (i.e. different type of appliance or accessory):

Yes

Specify: __________

No

NO

1. Do you have any new concerns about your ostomy?

YES (option/prompt to take picture here)

My concern(s) are (check all that apply):

Appliance Leakage

Appliance application technique

Odor

Stoma appearance (specify)

Skin (irritation, itching, pain)

Specify: __________

Increased output

Bleeding

Frequent appliance changes

Other: __________

NO

1. Are you participating in an ostomy support program sponsored by one of the ostomy supply companies?

YES

Specify: __________

NO

## Supplementary file 2: Modified Post-Study System Usability Questionnaire for Patients and Clinicians [22]

Please rate the following statements on a scale from 1-5 (1-strongly disagree, 2-disagree, 3-neutral, 4-agree, 5-strongly agree):

1. Overall, I am satisfied with how easy it is to use the how2trak application.

1 2 3 4 5 NA

1. It was simple to use the how2trak application.

1 2 3 4 5 NA

1. I was able to complete the tasks and scenarios quickly using the how2trak application.

1 2 3 4 5 NA

1. I felt comfortable using the how2trak application.

1 2 3 4 5 NA

1. It was easy to learn to use the how2trak application.

1 2 3 4 5 NA

1. I believe I could become productive quickly using the how2trak application.

1 2 3 4 5 NA

1. The how2trak application gave error messages that clearly told me how to fix problems.

1 2 3 4 5 NA

1. Whenever I made a mistake using the how2trak application, I could recover easily and quickly.

1 2 3 4 5 NA

1. The information (such as online help, on-screen messages, and other documentation) provided with the how2trak application was clear.

1 2 3 4 5 NA

1. It was easy to find the information I needed.

1 2 3 4 5 NA

1. The information provided was effective in helping me complete the tasks and scenarios.

1 2 3 4 5 NA

1. The organization of information on the how2trak application screens was clear.

1 2 3 4 5 NA

1. The interface of the how2trak application was pleasant.

1 2 3 4 5 NA

1. I liked using the interface of the how2trak application.

1 2 3 4 5 NA

1. The how2trak application has all the functions and capabilities I expected it to have.

1 2 3 4 5 NA

1. Overall, I am satisfied with the how2trak application.

1 2 3 4 5 NA

## Supplementary file 3: Patient Experience Survey[23,24]

We are interested in feedback about you and your experience of the care received after your hospital stay. Please answer all the questions yourself by circling the number that best applies to you. There are no 'right' or 'wrong' answers. The information that you provide will remain strictly confidential.

During your recovery, how would you rate the doctors you interacted with after you were discharged and left the hospital (1-poor, 2-fair, 3-good, 4-very good, 5-excellent):

1. Their knowledge and experience of your illness?

1 2 3 4 5 NA

1. The treatment and medical follow-up they provided?

1 2 3 4 5 NA

1. The attention they paid to your physical problems?

1 2 3 4 5 NA

1. Their willingness to listen to all of your concerns?

1 2 3 4 5 NA

1. The interest they showed in you personally?

1 2 3 4 5 NA

1. The comfort and support they gave you?

1 2 3 4 5 NA

1. The information they gave you about your illness?

1 2 3 4 5 NA

1. The information they gave you about your medical tests?

1 2 3 4 5 NA

1. The information they gave you about your treatment?

1 2 3 4 5 NA

1. The frequency of their visits/consultations?

1 2 3 4 5 NA

1. The time they devoted to you during visits/consultations?

1 2 3 4 5 NA

During your recovery, how would you rate the nurses you interacted with after you were discharged and left the hospital (1-poor, 2-fair, 3-good, 4-very good, 5-excellent):

1. The way they carried out your assessment ?

1 2 3 4 5 NA

1. The way they handled your care (gave you a plan, discussed options,…)?

1 2 3 4 5 NA

1. The attention they paid to your physical comfort?

1 2 3 4 5 NA

1. The interest they showed in you personally?

1 2 3 4 5 NA

1. The comfort and support they gave you?

1 2 3 4 5 NA

1. Their human qualities (politeness, respect, sensitivity, kindness, patience,…)?

1 2 3 4 5 NA

1. The information they gave you about your medical tests?

1 2 3 4 5 NA

1. The information they gave you about your care?

1 2 3 4 5 NA

1. The information they gave you about your treatment?

1 2 3 4 5 NA

1. Their promptness in answering your questions?

1 2 3 4 5 NA

1. The time they devoted to you?

1 2 3 4 5 NA

After your hospital stay, how would you rate the services and care organisation, in terms of (1-poor, 2-fair, 3-good, 4-very good, 5-excellent):

1. The exchange of information between health care professionals?

1 2 3 4 5 NA

1. The kindness and helpfulness of the technical, reception, laboratory personnel?

1 2 3 4 5 NA

1. The information you were given on your admission to the hospital?

1 2 3 4 5 NA

1. The information you were given on your discharge from the hospital?

1 2 3 4 5 NA

1. The waiting time for obtaining results of medical tests?

1 2 3 4 5 NA

1. The speed of getting medical tests and/or treatments?

1 2 3 4 5 NA

1. The ease of access (parking, means of transport, login…)?

1 2 3 4 5 NA

1. The ease of finding one’s way to the different departments?

1 2 3 4 5 NA

1. How would you rate the care received after your hospital stay?

1 2 3 4 5 NA

## Supplementary file 4: Informed Consent (attached)

## Supplementary file 5: Verbal Consent Script (attached)

## Supplementary file 6: Patient Information Sheet

Evaluating a Mobile Device Application for Wound Surveillance after Colorectal Surgery: Bringing Care Closer to Home

Thank you for helping us with the evaluation of a new mobile device application for the surveillance of wounds after colorectal surgery.

**What we ask of you:**

After your surgery, we kindly ask that you report your symptoms by completing a short survey online.

You will need to download the how2trak My Care application (app) on your phone, desktop, laptop or tablet (free). You can take photos of your wound and answer questions about your wound/health using this app.

**Downloading how2trak My Care**

*how2trak My Care* is free in the Apple or Android app store.

1. Go to your app store

2. Search for ‘how2trak My Care’ as your keyword


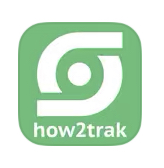


3. Download the app

4. Enter username and password, as below

Login Information

- Username = email (chosen by the participant)
- Password = auto-generated password (random mix of characters)

We ask that you photograph your wound and answer the questions in the application on **post-operative day 3, 5, 7, 10, 20 and 30**.

The purpose of the questions in the application are to help assess your incision and ostomy*, and see whether you need further medical attention.

If medically appropriate, the nurse will follow-up with you within 72 hours of completing the app questions. **If you have urgent concerns or this is an emergency, please present to the emergency department or call 911.**

If you have NON-urgent concerns regarding your incision or stoma, please indicate this in the app questions and the nurse will contact you.

*if applicable

## Supplement 7: Patient Application Response suggestive of surgical site infection:

(Based on the CDC criteria[5])

1. Is there more redness around your surgery incision?

-Yes

3. Do you have worse pain around your surgery incision?

-Yes

4. Is there drainage coming from your surgery incision?

-Yes

-More

5. The smell from your surgery incision is:

-Stronger

Picture (look for the following characteristics to suggest SSI)

-Localized swelling

-Erythema

-Drainage (purulent)
